# Cardiac manifestations of the coronavirus disease-19: a review of pathogenesis, clinical manifestations, diagnosis, and treatment

**DOI:** 10.11604/pamj.2021.39.173.27802

**Published:** 2021-07-06

**Authors:** Momina Khalid, Sana Awan, Nadia Nazir Jatoi, Hafsa Nazir Jatoi, Farah Yasmin, Rohan Kumar Ochani, Simran Batra, Farheen Malik, Jawad Ahmed, Sanchit Chawla, Ahmad Mustafa, Hassan Mehmood Lak, Salim Surani

**Affiliations:** 1 Department of Internal Medicine, Jinnah Sindh Medical University, Karachi, Pakistan,; 2 Department of Internal Medicine, Dow Medical College, Dow University of Health Sciences, Karachi, Pakistan,; 3 Department of Medicine, Cleveland Clinic Foundation, Cleveland, Ohio, 44111, United States of America,; 4 Department of Internal Medicine, Staten Island University Hospital, New York, United States of America,; 5 Department of Internal Medicine, Corpus Christi Medical Center, Corpus Christi, United States of America,; 6 Department of Internal Medicine, University of North Texas, Dallas, United States of America

**Keywords:** SARS-CoV-2, COVID-19, electrocardiogram, thrombolytic, myocarditis, troponin, IL-6 blockers, cardiovascular disease

## Abstract

The coronavirus disease-19 (COVID-19), first appearing in Wuhan, China, and later declared as a pandemic, has caused serious morbidity and mortality worldwide. Severe cases usually present with acute respiratory distress syndrome (ARDS), pneumonia, acute kidney injury (AKI), liver damage, or septic shock. However, with recent advances in severe acute respiratory syndrome-coronavirus-2 (SARS-CoV-2) research, the virus´s effect on cardiac tissues has become evident. Reportedly, an increased number of COVID-19 patients manifested serious cardiac complications such as heart failure, increased troponin, and N-terminal pro-B-type natriuretic peptide levels (NT-proBNP), cardiomyopathies, and myocarditis. These cardiac complications initially present as chest tightness, chest pain, and heart palpitations. Diagnostic investigations such as telemetry, electrocardiogram (ECG), cardiac biomarkers (troponin, NT-proBNP), and inflammatory markers (D-dimer, fibrinogen, PT, PTT), must be performed according to the patient´s condition. The best available options for treatment are the provision of supportive care, anti-viral therapy, hemodynamic monitoring, IL-6 blockers, statins, thrombolytic, and anti-hypertensive drugs. Cardiovascular disease (CVD) healthcare workers should be well-informed about the evolving research regarding COVID-19 and approach as a multi-disciplinary team to devise effective strategies for challenging situations to reduce cardiac complications.

## Introduction

Coronavirus disease-19 (COVID-19) was first discovered in December 2019 as an unusual pneumonia-like illness in Wuhan, China. Initially, not much was known about the disease, but the laboratory diagnosis of this infection later revealed the cause of the infection to be a new strain of *Coronaviridae* [1]. This virus was named the severe acute respiratory syndrome coronavirus-2 (SARS-CoV-2) due to its close resemblance to the previous strain that is severe acute respiratory syndrome coronavirus (SARS-CoV). Previously, Middle Eastern respiratory syndrome-CoV (MERS-CoV) and SARS-CoV strains of the same family have been known to cause serious morbidity and mortality in humans [1]. Genomic investigations of SARS-CoV-2 revealed a 79.6% similarity with the previous SARS-CoV strain [2]. Nevertheless, the biggest threat of this strain was its extremely high infectivity rate which caused the World Health Organization (WHO) to declare a global health emergency on January 30^th^, 2020 [1]. WHO defines R0 as the number of persons likely to get the infection from 1 infected individual and it was estimated to be up to 3.28 (1.4-6.49) for SARS-CoV-2 which was higher than for SARS-CoV [3]. The virus mainly spreads through human-to-human transmission via respiratory droplets, and as the incubation period ranges from 2-14 days, many asymptomatic cases continue to transmit the disease [1]. Regardless of all efforts to contain the disease, by 11^th^ March 2020, it had managed to spread across borders affecting 114 countries with more than 118,000 cases worldwide and consequently led WHO to declare it as a pandemic [1].

As of December 30^th^, 2020, 1,798,097 deaths have been reported with a total of 82,394,792 people infected around the world [4]. The clinical presentation in the initial months of the disease only constituted fever, fatigue, dyspnea, myalgia, and dry cough [1]; which also remained the basis of suspecting, testing, and diagnosing the illness [5]. Most critical patients presented with acute respiratory distress syndrome (ARDS) requiring respiratory support in the intensive care units (ICUs) [1]. However, as more was revealed about the new disease by different experiences of physicians across the world, it was discovered that the disease was not confined to respiratory symptoms but affected many other organs leading to cardiac, neurological, hematological symptoms as well as multi-organ dysfunction [6]. According to the recent data, serious cardiac manifestations after COVID-19 infection have been reported and resulted in an increased number of patients presenting to the ICUs. Patients with COVID-19 report various cardiac complications which include heart failure, increased troponin, and N-terminal pro-B-type natriuretic peptide levels (NTpro-BNP), cardiomyopathies, and myocarditis [7]. Although patients with diagnosed cardiovascular and congenital heart diseases are at a greater risk of developing serious COVID-19 complications, many studies now prove that COVID-19 has the potential to cause serious cardiac complications in previously healthy individuals [7].

Many viral infections have been associated with cardiac injuries particularly causing myocarditis [8]. Previous coronavirus strains such as MERS-CoV and SARS-CoV were also known to have implications on cardiac tissues [7]. Even though the mechanisms of cardiac injury due to SARS-CoV-2 remain uncertain, many authors suggest that cytokine storm, direct cardiac injury, and hypoxia of cardiac tissue might be the major contributors to serious complications [7]. Nonetheless, there is still plenty to be discovered and clinicians must understand the existing pool of information on cardiac complications to be able to prevent serious morbidity in those who can be saved. For cardiologists to vigilantly detect and treat potential COVID-19 patients presenting with cardiac symptoms, it is important to understand the mechanism, presentations, and learn about the treatments employed in such patients around the world. In this review we aim to explain the pathogenesis, risk factors of serious cardiac outcomes, and different proposed mechanisms of cardiac injury from COVID-19 infection, clinical presentations, and treatment options for critical patients with severe cardiac manifestations.

## Methods

For this review, a literature search was conducted using PubMed/MEDLINE and Google Scholar from its inception to December 2020. The following search string was used: (“SARS-CoV-2” or “COVID-19”) and (“cardiovascular diseases”) and (“pathogenesis” or “risk factors” or “diagnosis” or “management” or “treatment”). All articles in a language other than English were excluded.

## Current status of knowledge

**The cardiovascular incidence in COVID-19 patients:** the prevalence of existing cardiovascular diseases (CVD) in COVID-19 has not been reported to its utmost capacity majorly owing to the unsatisfactory testing, lack of extensive data, and biased sampling methods embraced towards hospitalized patients with serious comorbidities [9]. However, several studies have attempted to analyze the situation. The study conducted by Ruan Q *et al*. investigated the most likely cause of death amongst the critical COVID-19 patients and noticed that (n=22/68; 33%) people died of myocardial injury and respiratory failure. The number of patients dying due to isolated myocardial injury and/or heart failure was 7% [10]. Many studies have noticed a 5-7% rise in troponin levels in hospitalized COVID-19 patients [11, 12]. Whilst this rise may be mild to moderate in some cases, the rise suggesting myocardial injury is greatly associated with serious complications of the infection [13]. Moreover, Zhou *et al*. from Wuhan, China found that 28/54 (52%) of those who died due to COVID-19 had heart failure [14] and cases series from Seattle, USA reported heart failure amongst 9/21 (42.9%) of the patients [15]. There are many limitations in these findings because the definition of ‘heart failure´ considered in each study determines much of the generalization of the findings. It should also be noted that such numbers have not been seen in studies from Italy or in larger case series from China [9]. Many studies have highlighted the development of arrhythmias and sudden cardiac failure in COVID-19 patients and the first case series from Wuhan reported 16.7% to 44% increased arrhythmias incidence in hospitalized COVID-19 patients compared to those treated in ICUs as critical cases [9,13]. Lastly, another study concerning a group of patients (n = 120) with COVID-19 reported increased troponin levels (n = 12, 10%) and NT-proBNP levels (n = 33, 27.5%) [7].

**Potential risk factors for cardiovascular complications in COVID-19 patients:** most severe cases of COVID-19 have been observed in the elderly mostly due to diminished immunity or a greater burden of comorbidities [9]. Only a small percentage of cases have been reported in children potentially due to the same reasons [9]. Hence, the age for one seems to be playing a major role in the development of serious cardiovascular complications in COVID-19 patients [9]. Cardiovascular risk factors that affect immunity, including diabetes mellitus and hyperlipidemia, lead to dysregulated immunologic status and increases the risk of CVS complications among COVID-19 patients. Although data may vary considerably across different regions of the world, rates of comorbidities such as hypertension, diabetes mellitus, and coronary artery disease have been higher in patients presenting with serious complications of COVID-19 in most studies [16]. A meta-analysis pooling 6 studies inclusive of a total of 1527 patients further emphasizes the afore-mentioned point as the study pointed out that hypertension (17.1%), cerebrovascular and cardiac diseases (16.4%), and diabetes mellitus (9.7 %) were the most common comorbidities in COVID-19. It further reported that the presence of cardio-cerebrovascular disease resulted in a 3-fold greater risk of severe disease or requiring ICU admission [17]. Three studies conducted with a total of 1,278 COVID-19 patients, to compare the prevalence of hypertension, cardiac and cerebrovascular disease, and diabetes mellitus in severe and non-severe patients, reported that 28.8% of the severe cases and 14.1% of non-severe cases had hypertension [9].

Similarly, 16.7% of severe cases and 6.2% of non-severe cases had the cardiac and cerebrovascular disease [9] while the cases with diabetes mellitus included 11.7% of severe cases and 4.0% of non-severe cases [9]. Another case series of 5700 patients in New York, USA has shown that the most common comorbidities in COVID-19 patients included hypertension (n=3026; 56.6%), obesity (n=1737; 41.7%), and diabetes mellitus (n=1808; 33.8%) that increases the risk of CVS complications [18]. Furthermore, the median score on the Charlson Comorbidity Index was estimated to be 4 points (Interquartile range {IQR}, 2-6), which refers to a 53% estimated 10-year survival and shows that the burden of comorbidities is high in these patients [18]. Furthermore, plasma glucose levels and diabetes mellitus have been reported to be independent predictors of death and morbidity in patients with SARS-CoV-2 [19]. Due to the already established relationship of serious cardiac complications with previous lethal strains of the coronavirus, it is not surprising that SARS-CoV-2 appears to affect cardiac tissues similarly, especially in patients with pre-existing cardiac compromise. Moreover, a further study inclusive of 44,672 patients with COVID-19 linked patients with CVD to a 5 times higher case fatality rate (10.5%) compared to those patients with no history of CVD (2.3%) [20]. Furthermore, there is an increase in expression of ACE-2 receptors in hypertensive patients because ACE-2 is an important part of the counter-regulatory pathway of the renin-angiotensin-aldosterone system (RAAS) which functions to reduce blood pressure, inflammation, and fibrosis and hence plays a prominent role in the pathophysiology of hypertension [21]. However, higher expression of ACE- 2 receptors in patients with hypertension has been reported to increase their susceptibility to SARS-CoV-2 infection [9]. Other risk factors for cardiovascular complications include previously diagnosed CVD, immune activation, shock, metabolic disarray, coagulopathy, and immobility among COVID-19 patients.

**Figure 1:**
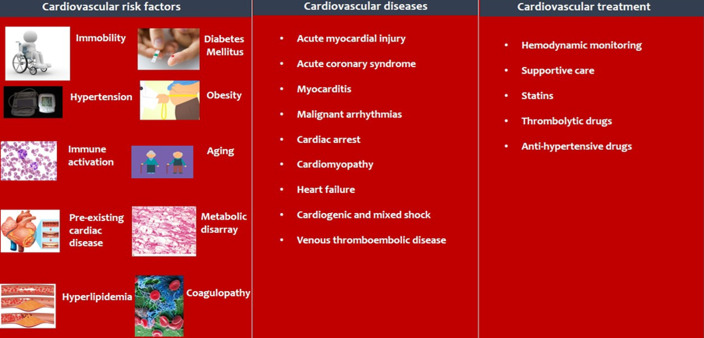
the cardiovascular impact of the COVID-19 pandemic

**Cardiovascular diseases in COVID-19 patients:** based on currently observed disease patterns, the cardiovascular impact of COVID-19 is demonstrated in Figure 1.

Acute myocardial injury, myocarditis, and acute coronary syndrome: acute myocardial injury is defined as an elevated cardiac troponin (cTn) with at least one value above the 99th percentile upper reference limit [22]. It may occur as a result of myocardial ischemia or non-ischemic myocardial processes such as myocarditis [22], pulmonary embolism, sepsis, severe respiratory infection with hypoxia, and cardiac adrenergic hyperstimulation in cytokine storm syndrome [23]. Overall, acute myocardial injury is the most commonly reported cardiovascular manifestation in COVID-19, having an incidence of roughly 8-12% in the positive cases [24] and evidence now suggests a correlation of elevated cTn levels with a poor prognosis in COVID-19. In a retrospective cohort study of 416 hospitalized COVID-19 patients from China, 19.7% (82 patients) were reported to have a myocardial injury with an in-hospital mortality rate of 51.2% (42/82), where higher mortality rates were reportedly more frequent in those with significantly elevated cardiac Troponin I (cTnI) levels [25]. A similar retrospective study from China also noted significantly elevated cTnI levels throughout the clinical course of non-survivors, with further increases noted as their clinical condition worsened [14]. Consistent with these findings, Guo *et al*. [26] reported in a case series of 187 patients, 27.8% (52 patients) demonstrated myocardial injury, as determined by elevated Troponin T levels (TnT), with an in-hospital mortality rate of 59.6% (31/52). Importantly, a significantly high mortality rate of 69.4% (25/36) was documented in those with elevated TnT levels alongside underlying cardiovascular disease while a mortality rate of 37.5% (6/16) was observed in those with elevated TnT levels without previous history of CV disease [26]. Besides, patients with elevated TnT levels were noted to have elevated C-reactive protein and NT-proBNP levels, suggesting a likely correlation between myocardial injury and the degree of inflammation [26].

The exact mechanism of myocardial injury in COVID-19 is still being explored but the findings can be explained with the help of literature from influenza [27, 28] and other acute inflammatory conditions [29] which shows that patients with risk factors for atherosclerotic disease and/or those with long-term coronary artery disease have a greater risk of developing acute coronary syndrome during acute infections. This may be attributed to the myocardial ischemia or infarction precipitated by infections as a result of the myocardial oxygen demand/supply imbalance, the combined effects of vascular inflammation and prothrombotic endothelial dysfunction may also contribute to the ischemic elevation of troponin in COVID-19 [23]. It is also believed that the cytokines released during severe systemic inflammatory stress could lead to atherosclerotic plaque disruption [26]. Moreover, infection of endothelial cells in heart vessels with no evidence of lymphocytic myocarditis, reported in a case report, could suggest an additional mechanism of troponin elevation and myocardial injury in COVID-19 [30]. COVID-19 is predominantly known for its presentation as a respiratory illness, however, there have been reports of cases that first presented with cardiovascular manifestations and were later diagnosed with COVID-19. One such case includes a 37-year-old male presenting with chest pain, dyspnea, and accompanying diarrhea. Electrocardiogram (ECG) reported ST-segment elevation, but computed tomography (CT) coronary angiography revealed no coronary obstruction. An enlarged heart with a left ventricular ejection fraction (LVEF) of 27% and left ventricular end-diastolic diameter of 58 mm was noted on the echocardiogram. Cardiac biomarkers were elevated with a cTnT >10,000 ng/L and natriuretic peptide BNP >21,025 ng/L. The patient was ultimately diagnosed with coronavirus fulminant myocarditis with cardiogenic shock and pulmonary infection. Therapy with intravenous immunoglobulin and methylprednisolone resulted in the normalization of cardiac biomarkers and ejection fraction within 3 weeks [31].

**Figure 2:**
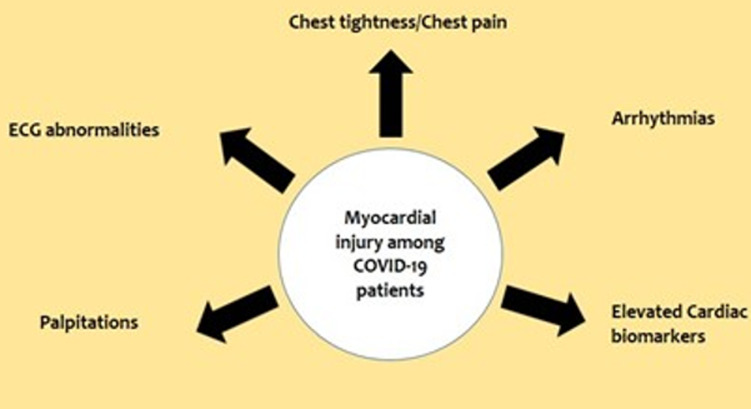
clinical manifestations of COVID-19 associated myocardial injury

Similarly, a case report from China observed a 63-year-old male with no previous cardiac history or hypertension presenting with fever, shortness of breath, and chest tightness after activity. Chest radiographs revealed ground-glass opacities typical of viral pneumonia and were confirmed as COVID-19 positive via sputum testing. Electrocardiogram showed sinus tachycardia with no ST-segment elevation, while echocardiography revealed enlarged left ventricle (61 mm), diffuse myocardial dyskinesia, LVEF of 32%, and pulmonary hypertension (44 mmHg). Markers of myocardial injury were elevated with cTnI >11.37 g/L and NTBNP >22,600 pg/ml. The patient was diagnosed with severe pneumonia, ARDS, and fulminant myocarditis. To reduce cardiopulmonary burden, extracorporeal membrane oxygenation (ECMO) was used and therapy with immunoglobulin, methylprednisolone, antivirals, and continuous renal replacement (CRRT) was initiated. LVEF recovered to 68%, left ventricle, and wall thickness returned to normal ranges with treatment. However, infection-related markers increased, pulmonary artery systolic pressure (PASP) and tricuspid annular plane systolic excursion (TAPSE) declined, septic shock and disseminated intravascular coagulation (DIC) developed and the patient died on day 33 of hospitalization [32]. Furthermore, observation of 419 cases of COVID-19 revealed elevated Troponin I in 32 (8%) cases and per the echocardiographic findings, 2 cases were diagnosed with fulminant myocarditis [32]. There have also been similar reports from other countries where a small subset of patients presented with chest pain and abnormal electrocardiograms, without any evidence of coronary stenosis, eventually testing positive for COVID-19 [33]. Therefore, these observations help conclude that myocardial injury is a potential complication of COVID-19 and can have a significant impact on its outcome. A noteworthy observation seen in the largest COVID-19 case series of 72,314 cases, recently published by the Chinese Center for Disease Control and Prevention, revealed an overall case fatality rate (CFR) of 2.3%, out of which 10.5% of deaths were seen in patients with pre-existing CVD [34]. A summary of the clinical manifestations observed in COVID-19 related myocardial injury is given in Figure 2.

**Cardiac arrhythmia and cardiac arrest:** cardiac arrhythmias are another frequent facet of cardiac involvement in COVID-19. In a retrospective observational study of 187 patients from Wuhan, patients with elevated TnT levels were more likely (11.5%) to develop malignant arrhythmias, including ventricular tachycardia or fibrillation [26]. Although not a frequent manifestation, 7.3% of patients presented with heart palpitations in another study of 137 COVID-19 patients from Wuhan [35]. Of note, cardiac biomarkers should be assessed in all patients self-isolating at home, and even after hospital discharge, the myocardial injury may cause atrial or ventricular fibrosis consequently leading to cardiac arrhythmias [36]. Reports from the National Health Commission of China have shown cardiac arrests, elevated cardiac biomarkers, and abnormal ECG findings even in patients without underlying CVD [37]. Besides, hypoxemia in COVID-19 is important to monitor as it may trigger atrial fibrillation, the most common arrhythmia in the elderly population [38]. Drug-induced myocardial damage is also of concern in COVID-19 treatment as antivirals can cause arrhythmias and cardiac insufficiency [37].

**Cardiomyopathy and heart failure:** earlier reports investigating case fatality rates in COVID-19 patients, attributed heart failure as the cause of death in 49% of patients that exhibited significantly elevated levels of troponin and natriuretic peptides [39]. In a case series of 191 positive cases, 23% (44 patients) developed heart failure and was more common in non-survivors (52%) in comparison to those who survived (12%) [34]. Severe acute heart failure has also been a manifestation of COVID-19 in some case reports [40]. Whether heart failure occurs due to exacerbation of underlying structural heart disease, myocarditis, or stress cardiomyopathy is still unclear. However, cytokine-related myocardial dysfunction in the progressive stages of COVID-19 is postulated [41]. Thus, it may be considered that heart failure plays a prominent role as a risk factor for severe disease and increased mortality in COVID-19.

**Cardiogenic and mixed shock:** among the various reported cardiovascular presentations in COVID-19, the case of a 69-year-old patient developing respiratory distress and cardiogenic shock are of importance as this is the first reported case directly linked to the myocardial localization of SARS-CoV-2 [42]. Initial echocardiography revealed a dilated as well as diffuse left ventricle hypokinesia with an ejection fraction of 34%, which later dropped to 25%. Venous-arterial extracorporeal membrane oxygenation (VA-ECMO) implantation was instituted and later upgraded to venous-arterial-venous ECMO. Left ventricle function recovered to normal levels however, on day 12 the patient died of gram-negative pneumonia and septic shock. As per protocol in non-ischemic cardiogenic shock, an endomyocardial biopsy was performed, which demonstrated low-grade myocardial inflammation and viral particles in the myocardium having morphology and size of coronaviruses, suggesting either viremia or infected macrophage migration from the lung [42]. Shock is reported as a common complication in the ICU of COVID-19 patients. A study of 113 deceased COVID-19 patients revealed that 41% of patients developed shock [39], while in another study of 138 COVID-19 patients, 8.7% developed shock [11]. Similarly, in a study on 41 COVID-19 patients, 23% in ICU care developed shock [12]. Hence, it is essential to have a low threshold for considering cardiac involvement, as cardiac and respiratory symptoms may overlap and even be masked. ARDS in COVID-19 is manifested as hypoxemia and ground-glass opacities on imaging [43] and similar manifestation can be seen in cardiogenic pulmonary edema, whether coexisting or de novo, thus, making it important to assume cardiogenic or mixed cardiac and pulmonary causes in COVID-19. It is crucial to ascertain whether an associated cardiogenic component is present when considering ECMO for mechanical respiratory and circulatory support, as this may lead to a difference in the selection of a device (venovenous or venoarterial ECMO cannulation) [9].

**Venous thromboembolic disease:** commonly, inflammatory states have a greater risk of venous thromboembolism (VTE), and likely, COVID-19 patients with respiratory failure, underlying comorbidities, and prolonged bed rest will be at an increased risk of VTE. Data from 184 patients with COVID-19 pneumonia shows the incidence of thrombotic complications to be as high as 31% in the ICU, with pulmonary embolism being the most frequent thrombotic complication (81%), seen in 25 cases [44]. In a study evaluating the risk of VTE in 138 COVID-19 patients, 15 were critically unwell and had a higher risk for VTE in comparison with the non-critical patients [45]. Similarly, another study of 81 COVID-19 positive cases reported that 20 patients developed lower extremity VTE, of which 8 patients died. Risk factors for VTE include older age, elevated D-dimer levels, and low lymphocyte levels [46]. In terms of laboratory findings, D-dimer >1µg/ml was associated with a fatal outcome in COVID-19 [14]. Markedly raised D-dimer concentrations were reported in 35% of a deceased patient in comparison to only 2% of 150 recovered patients [39]. In another study, COVID-19 non-survivors had significantly high D-dimer and fibrin degradation product levels, and 71% met the clinical criteria for disseminated intravascular coagulation (DIC) during their disease [47]. Mechanisms contributing to these findings include systemic pro-inflammatory cytokine responses causing plaque disruption and induction of pro-coagulation factors predisposing to thrombosis and ultimately ischemia [29,48]. Vascular inflammation with endotheliitis may also lead to the development of a hypercoagulable state and DIC [34]. Endotheliitis in the setting of COVID-19 has been reported in some instances. A study examining 7 lungs obtained during the autopsy of COVID-19 patients found severe endothelial injury associated with the presence of intracellular SARS-CoV-2 virus, along with widespread thrombosis, microangiopathy, and occlusion of alveolar capillaries [49]. Additionally, endotheliitis has also been seen to involve the heart, kidney, liver, and small intestine in COVID-19 patients [30].

A retrospective study of 97 patients with severe COVID-19 noted that patients who received prophylactic low-molecular-weight heparin in the setting of sepsis-induced coagulopathy had lower mortality rates [50]. However, caution is needed as the thrombo-prophylactic regimen for hospitalized COVID-19 patients is still unclear. Though proinflammatory and prothrombotic states are frequently reported in severe COVID-19 cases, coagulopathy has been documented in one-fifth of cases [14]. Thus, careful monitoring for thrombotic complications as well as bleeding is needed. Moreover, older age increased d-dimer levels, and a raised Sequential Organ Failure Assessment (SOFA) score can help in the early identification of severe cases [14].

**Impact of the COVID-19 pandemic on patients with underlying cardiovascular diseases:** COVID-19 initially thought to impact the respiratory system only continues to show heterogeneous clinical manifestations. However, the presence of comorbidities in such patients escalates the risk of poor prognosis and significantly increases the disease severity. Although people with comorbidities are highly vulnerable to develop COVID-19 and experience severe symptoms, those with pre-existing CVD have been reported to have the highest fatality rate among all COVID-19 patients.

Prevalent CVD especially among people of older age and diabetics is a marker of immunologic aging and dysfunction and relates to poor COVID-19 prognosis. In a cohort study of 191 patients from Wuhan, China, CVD was found in 8% of patients (13% of non-survivors) whereas another cohort of 138 hospitalized patients with COVID-19 showed CVD in 15% (25% in patients requiring an ICU) suggesting the prognostic impact of this comorbidity [51]. Furthermore, patients awaiting or having undergone heart transplantation represent an especially vulnerable group as COVID-19 poses a challenge for heart transplantation, affecting donor selection, immunosuppression, and post-transplant management. A survey of 87 heart transplant recipients in Wuhan, China, did not find a higher risk of infection with SARS-CoV-2 if routine preventive measures were used; however, this finding needs to be confirmed in larger populations [51].

Patients with underlying CVD, when infected with SARS-CoV-2 are more likely to develop an unstable hemodynamic status, placing a higher workload on the ventricles. This can exacerbate the already existing ventricular dysfunction that can cause cardiogenic shock. Furthermore, the systemic inflammation with local inflammatory infiltration caused by COVID-19 can result in the development of a hypercoagulable state and atherosclerotic plaque rupture which may culminate in thrombotic events. A study conducted during the first phase of the COVID-19 pandemic showed a significant decline in cardiovascular hospitalizations in Massachusetts, USA. A similar scenario was seen in Italy where a nearly 50% reduction in admissions for acute coronary syndromes was reported during the COVID-19 surge, compared with a similar time frame in 2019 [52]. This is mainly due to high-risk patients with the acute cardiovascular illness being restricted to visit the hospital to avoid nosocomial viral transmission which might predispose such patients to detrimental clinical outcomes.

**Diagnosis of cardiovascular complications in COVID-19 patients:** COVID-19 although predominantly a respiratory illness, can present with chest tightness and heart palpitations as the first signs, and keeping in mind the significant impact of cardiovascular involvement on the outcome of COVID-19 patients, the importance of early identification cannot be understated. Therefore, cardiovascular assessment is advised for all patients presenting with associated cardiovascular risk-factors, pre-existing CVD, cardiovascular signs and symptoms, ECG alterations, elevated cardiac biomarkers, or those requiring hospitalization [26]. However, given the highly contagious nature of SARS-CoV-2, it is important to reduce staff and patient exposure where necessary by limiting diagnostic modalities to the one directly influencing patient management [53]. For in-hospital cardiovascular testing, telemetry should be used in all cases, especially in critical patients [54].

Baseline ECG should be obtained at the time of admission and repeated daily in severe COVID-19 infection as it may identify new-onset arrhythmias as seen in a small subset of ICU patients with COVID-19 [11,55]. Besides, ECG monitoring of patients receiving hydroxychloroquine and azithromycin therapy is necessary due to their association with QTc interval prolongation [56]. In a study of 201 COVID-19 patients treated with chloroquine/HCQ ± azithromycin, the maximum corrected QT interval while receiving treatment was significantly prolonged in the combination therapy group (470.4 ± 45.0 ms) in comparison to the monotherapy group (453.3 ± 37.0) [57], while in a study of 196 COVID-19 patients receiving HCQ and azithromycin, 10.2% patients developed a QTc ≥ 500 ms [58]. Cardiac troponin and NT-proBNP levels should be measured at the time of admission, with regular monitoring if found elevated. Troponin rise is usually a late manifestation in severe COVID-19 and markedly elevated levels are associated with significant in-hospital mortality. It is recommended to avoid routine transthoracic echocardiogram (TTE) in COVID-19 patients unless abnormal findings are noted on point-of-care-ultrasound (POCUS) [54]. The use of echocardiography and vascular ultrasound should also be implemented in severe COVID-19 patients. In L-type dyspneic COVID-19 patients, i.e. having preserved compliance, the cardiac ultrasound may show ventricular interdependence caused by greater respiratory effort and show a diastolic ventricular septal shift with left ventricular hypo-diastole and decreased stroke volume.

In contrast, in H-type COVID-19 patients, i.e. having high pulmonary elastance, that are on positive-pressure mechanical ventilation, the cardiac ultrasound may reveal myocardial injury directly linked to mechanical ventilation [59]. Use of cardiac Magnetic Resonance Imaging (MRI) may also prove beneficial as findings from a cohort study conducted on 100 patients recently recovered from COVID-19 found cardiac involvement in 78% of patients and continued myocardial inflammation in 60% of patients [60]. Moreover, critically ill COVID-19 patients are at an increased risk of VTE, therefore monitoring of coagulation markers is essential as a D-dimer >1 µg/ml is associated with a poor outcome. The use of vascular ultrasound in combination with compression ultrasonography can help identify the presence of deep venous thrombosis in mechanically ventilated patients [59]. The use of neutrophil to lymphocyte ratio (NLR) and platelet to lymphocyte ratio (PLR) as a diagnostic and prognostic marker has been found to improve the prediction of COVID-19 disease severity. A retrospective study found NLR to be a superior prognostic parameter for progression from mild to severe illness at an optimal threshold of 3.3 and the largest area under the curve (AUC) [61]. Likewise, another study of 222 COVID-19 patients recorded disease severity rates to be the highest (72.3%; 34/47) among patients with high NLR and high IgG levels. In addition, treatment with mechanical ventilation was also highest (44.1%) among the high NLR and high IgG level patients, while recovery rates were lowest (58.8%; 20/34) [62]. In a meta-analysis of 6320 COVID-19 patients, elevated WBC count, neutrophil count, prothrombin time, D-dimer, fibrinogen, ESR, procalcitonin, IL-6, and IL-10 were all found to be associated with severe disease, ICU admission, and poor outcome in COVID-19 [63]. The diagnostic approaches for identifying cardiovascular complications in severe and non-severe cases are illustrated in Table 1 [51].

**Table 1:** diagnosis of cardiovascular complications

Non-severe cases	Severe cases
Telemetry	Telemetry
ECG(on admission)	ECGs(daily)
Cardiac biomarkers: Troponin, NT-proBNP (on admission)	Cardiac biomarkers: Troponin, NT-proBNP (daily)
Inflammatory markers: CRP, Ferritin, IL-6 (on admission)	Inflammatory markers: CRP, Ferritin, IL-6 (Q.O.D if ICU)
Coagulation markers: D-dimer, Fibrinogen, PT, PTT (on admission)	Coagulation markers: D-dimer, Fibrinogen, PT, PTT (QOD if ICU)
No routine TTE(POCUS indicated only if Shock, new HF, new persistent arrhythmia, ECG changes, Tn >200NG/L, ScvO2 <60%)	POCUS and 12 lead ECG (if Tn >200 ng/L, ScvO2 <60%)
	Formal TTE and cardiology consult if POCUS and 12 lead ECG abnormal (new decline in LVEF <50%)
	Advanced CV imaging: pharmacologic nuclear stress testing, TEE, cardiac CT, coronary CTA, cMRI)

**Abbreviations:** ECG, electrocardiogram; NT-proBNP, N-terminal-pro hormone brain natriuretic peptide; CRP, C-reactive protein; IL-6, interleukin 6; Q.O.D, every other day; PT, prothrombin time; PTT, partial thromboplastin time; TTE, transthoracic echocardiogram; POCUS, point-of-care-ultrasound; HF, heart failure; Tn, troponin; ScvO2, central venous oxygen saturation; CV, cardiovascular; cardiac CT, cardiac computed tomography; CTA, coronary computed tomography angiography; cMRI, cardiac magnetic resonance imaging.

**Figure 3:**
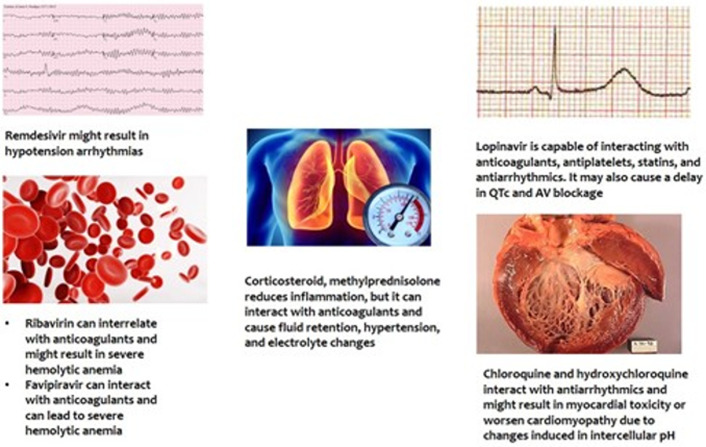
adverse cardiovascular effects of COVID-19 drugs

**Treatment and management of cardiovascular complications in COVID-19 patients:** as of now, vaccine or disease-specific treatment for SARS-CoV-2 is unavailable but new drugs as well as combinations of drugs like remdesivir, lopinavir-ritonavir, or lopinavir-ritonavir and Interferon Beta-1b, are being tested for use in severe cases [64]. However, it has been highlighted that some of the drugs being used in treatment for COVID-19 have adverse cardiac effects. Furthermore, most of the drugs that are studied have been found interacting with cardiovascular drugs including antihypertensive, antiarrhythmic, anticoagulants, and antiplatelet [20]. The drugs under investigation now include antivirals, antimalarial, azithromycin, corticosteroids, and biologics [20]. Some of these drugs such as INF and Ribavirin were used previously in the MERS outbreak for severely ill patients [65]. The use of some of these may have side effects. Few of these drugs along with their effects are mentioned in Figure 3 [20].

Hence, emergency health care workers must be made aware of these potential complications that may surface during the treatment of COVID-19 patients [20]. Currently, the best available management option for patients with CVD in SARS-CoV-2 high transmission areas is hemodynamic monitoring and the provision of supportive care while also trying to minimize the spread of the disease [66]. Furthermore, since it has been previously established that there is an elevation of inflammatory markers and a cytokine storm with high Interleukin-6 (IL-6) in severe COVID-19 patients, medications like tocilizumab and sarilumab are being investigated that could potentially reduce this effect by blocking the IL-6 for treatment in severe cases [66]. However, recent research indicates no potential benefit of IL-6 blockers [67]. Also, as is already understood that the use of statins produces vascular and myocardial anti-inflammatory effects and observational studies provide evidence that statin-treated patients have better cardiovascular outcomes. Hence, they could also be used to treat patients with CVS complications, however, a few COVID-19 patients may develop rhabdomyolysis or acute liver injury in which case statin must immediately be terminated. Another concern with the use of statins is that in the case of the use of lopinavir/ritonavir treatment by the patient, the dose of statins must be reduced or substituted as lopinavir/ritonavir can interact with statins [68]. The use of thrombolytic in reperfusion therapy is also amongst the best available options. The fibrin-specific drug, tenecteplase is a reliable option to treat patients with COVID-19 [69].

Most hospitalized patients have underlying comorbidities such as hypertension and, in these patients, LV hypertrophy or heart disease may occur in which case constant monitoring of potassium levels is important. If patients become hypotensive, antihypertensive therapy might need to be discontinued. On the other hand, for hypertensive patients, antihypertensive drugs are infrequently required to maintain the recently ventilated patient´s blood pressure below levels of grade 2 hypertension (BP > 160/100 mmHg) [68]. Finally, for the effective management of COVID-19 patients with underlying CVD, health care workers must prevent under-diagnosis of CVD by suspecting potential acute myocardial infarction or heart failure in COVID-19 patients [66].

**Proposed considerations for cardiovascular health-care workers during COVID-19 pandemic and management of cardiovascular diseases:** as COVID-19 cases continue to emerge, it is evident that affected individuals with pre-existing CVD have an increased risk of developing cardiovascular complications and death. Hence, all CVD healthcare workers need to stay updated with evolving information and new research, closely collaborate with pulmonologists, infectious-disease experts, surgeons, pharmacists, and hospital leadership, among others to come up with effective strategies for challenging situations [9]. In a report of 138 confirmed COVID-19 cases, 41.3% were considered to have acquired the infection from the hospital, and more than 70% of these patients were health care professionals (HCPs) [68]. A similar situation was reported from China where 1716 of the 44672 (3.8%) infected individuals were HCPs [68]. For this purpose, both WHO and Centers for Disease Control and Prevention (CDC) have issued personal protective equipment (PPE) guidelines where they recommend taking standard precautions such as the use of face mask, eye protection, gown, gloves, and respirators if available especially when performing percutaneous invasive procedures like coronary angiography, percutaneous coronary intervention (PCI) and electrophysiology (EP) procedures [9]. It is recommended for all HCPs to monitor their health status and cease patient care activities in case symptoms develop. Furthermore, a nasopharyngeal swab test and self-isolation are deemed necessary to restrict transmission.

Relating to patient care it is advised that any suspected COVID-19 patient recently admitted to the cardiology ward or brought to the emergency to be tested, regarded as COVID-19 infected, and managed with all the relevant precautions [68]. It is also important to reevaluate the transportation of patients to the catheterization laboratory for procedures as it carries the risk of contamination, therefore whenever possible, procedures at the bedside should be considered, such as intra-aortic balloon pump insertion. In the case of ST-elevation myocardial infarction (STEMI) patients, primary percutaneous coronary intervention (PPCI) is the gold standard with 30-day mortality which is much lower than those treated with thrombolysis [70]. However, in the setting of a pandemic, PPCI pathways may be delayed during the pandemic by up to 60 minutes, but a maximum delay of 120 minutes should remain the target. Fibrinolysis can be considered as first-line treatment if this target time is likely to be breached and there are no contraindications [71]. In addition, to minimize ICU bed utilization there might be a need to preferentially consider cardiac surgery or percutaneous interventional approaches for urgent scenarios that cannot wait (e.g. percutaneous coronary intervention rather than coronary artery bypass graft surgery or transcatheter valve solutions rather than surgery).

Given the rise in cases that can potentially exceed the hospital´s capacity, it is imperative to develop specific protocols to effectively use resources for CV patients such as postponing elective procedures, keeping in view of preserving limited inpatient resources for severe and high-risk CV patients. Telemedicine such as telephone, video-based software, or virtual clinics must be arranged to avoid nonessential interactions between both patients and healthcare workers to mitigate transmission risk. The patients must be counseled about promptly reporting new symptoms, safe physical activity options, and access to healthy food choices whereas the implementation of adaptive strategies for cardiac rehabilitation is recommended, including home-based cardiac rehabilitation, to ensure continuity of this essential service [72]. This approach has already been implemented in many countries [70]. Lastly, this is a challenging time for all HCPs battling the pandemic, which includes the increased burden of illness, increased risk for infections, and adverse effects on mental health. A study reviewed the psychological effects of quarantines during disease outbreaks. The report takes information from another study published in 2004 during the SARS-CoV outbreak, which revealed that quarantined hospital staff was more likely to report exhaustion and irritability, anxiety, and depression [73]. Therefore, all HCPs are encouraged to develop self-care strategy that will enable them to carry on their duties such as the recommendations provided by the US Department of Veteran Affairs for healthcare providers which include working in partnerships or teams, regularly seeking out accurate information and mentoring to assist in making decisions and time-outs for basic bodily care and refreshments [74].

## Conclusion

COVID-19 patients with cardiovascular complications constitute a great bulk of the admissions to the ICUs, as well as those requiring intubation, mechanical ventilation and ultimately progressing to fatal outcomes. Therefore, it is crucial to risk-stratify patients with pre-existing CVD at the time of admission. Moreover, it is necessary that all physicians belonging to the COVID-19 management team be vigilant of the cardiovascular implications of COVID-19 to rapidly identify and manage the clinicopathological signs as this may not only assist in the early prediction of disease severity but possibly improve the survival rates of COVID-19 patients. It is also recommended that a cardiologist be a part of the COVID-19 care team to guide effective treatment methodologies.

### What is known about this topic

- Most critical patients presented with acute respiratory distress syndrome (ARDS) requiring respiratory support in the intensive care units;
- There is a already established relationship of serious cardiac complications with previous lethal strains of the coronaviruses i.e. MERS and SARS-CoV;
- Although the mechanisms of cardiac injury due to SARS-CoV-2 remain uncertain, many authors suggest that cytokine storm, direct cardiac injury, and hypoxia of cardiac tissue might be the major contributors to serious complications.

### What this study adds

- Risk factors for cardiovascular complications include previously diagnosed CVD, immune activation, shock, metabolic disarray, coagulopathy, and immobility among COVID-19 positive patients;
- Cardiologists must stay updated with evolving information and new research, closely collaborate with pulmonologists, infectious-disease experts, among others to devise effective strategies to reduce mortality and morbidity in COVID-19 positive patients with pre-existing cardiovascular diseases;
- It is crucial to risk-stratify COVID-19 positive patients with pre-existing cardiovascular disease as they are at a greater risk of developing adverse complications and higher fatality rates.
